# Preliminary Report Concerning the Post Mortem Changes in the First Person Executed by Electricity

**Published:** 1890-10

**Authors:** E. C. Spitzka

**Affiliations:** New York


### PRELIMINARY REPORT CONCERNING THE POST MORTEM CHANGES IN THE FIRST PERSON EXECUTED BY ELECTRICITY.

BY DR. E. C. SPITZKA, NEW YORK.

Translated by M. B. Hutchins, M. D.

William Kemmler, executed by means of a Westinghouse Dynamo, was apparantly dead, in the usual sense of the word, after the first passage of the current. All contrary statements, which unfortunately are sustained by the majority of the physicians present, rest upon a truly fearful ignorance of the most rudimentary physiology, and the usual symptoms of death. When the current was suspended Kemmler remained stationary—in tetanus; after a minute the muscular contraction relaxed, and collapse of the thorax led to the escape of air through the mucus, quite thickly collected in the larynx. Careful observation showed that the so-called appearance of respiration was nothing other than collapse of the air vessels. Pulse was not present. That places rendered pale through pressure again became red could be established an hour after the brain, cord and contents of the thorax and abdomen had been removed. That blood flowed from the hand, injured through spasmodic contraction, and pressure of the nails, is true. So flowed blood from every place selected for section, as it indeed possessed the noteworthy fluid character, which is also observed in cases of accidental death from the electric current.

The face had an extremely natural expression, as often occurs in cases of sudden death. The electric tetanus of the abdominal muscles had not ceased an hour after death, and apparantly passed directly over into *rigor mortis*, while a stage of relaxation in the extremities, of about ten minutes' duration, preceded. In general, *rigor mortis* visibly, gradually extended from above downwards, and attained a more marked degree in the neck and upper extremities than in the lower. Unfortunately the tendon reflex was not tested in the interval between the two "shocks." Immediately after the passage of the second current these failed.

So deficient were the preparations for the physicians' examination (writer concerned only with the nervous system) that the taking of the temperature did not occur until two and one-half hours after death. The writer demonstrated an actually *burning* heat upon a space, upon the back of the neck, six centimetres in size. The temperature taken by means of an ordinary clinical thermometer, two and one-half hours after death, was 99½° F. and 97½° ten minutes later. *Six hours after death*, the thermometer passed into the fourth ventricle showed a temperature, still, of 96½° F., at a room temperature of 83° F. The pons and medulla-oblongata felt strikingly warm in contrast to the remaining, "cooled off" portions of the brain. This marked contrast was established on all sides. (I here pass over the usual appearance of the internal organs, as likewise the description of the burn-eschar, which I have presented in another place, in order to briefly describe the brain alterations.)

Corresponding to the point where the head electrode had been employed, the brain appeared pale through the dura mater. It was also extremely anæmic in the "precentral" parts and in those adjacent to the longitudinal fissure. In this region, and its borders, I found, upon transverse section, the following noteworthy alterations—gradually passing over into normal: The outer layer . . . . was harder and separated from the deeper, normally colored, cortex by a brittle, as if cariously broken down layer. The surface of the fourth ventricle was the seat of four, quite important, extravasations, proceeding from the branches of the smaller vessels. To me the pons seemed somewhat soft, in comparison with others similarly examined in fresh condition. That an intense caloric development was present in the vicinity of the "pons-oblongata" (?) sufficient to pass through the soft portions, is to me fully as striking as an inexplicable fact. More complete anatomical descriptions are reserved.—*Medicinische Monatsschrift.*

